# PI3K p85α Subunit-deficient Macrophages Protect Mice from Acute Colitis due to the Enhancement of IL-10 Production

**DOI:** 10.1038/s41598-017-06464-w

**Published:** 2017-07-21

**Authors:** Shusaku Hayashi, Takayuki Hamada, Donald G. A. Zinsou, Momoe Oshiro, Kana Itoi, Takeshi Yamamoto, Makoto Kadowaki

**Affiliations:** Division of Gastrointestinal Pathophysiology, Institute of Natural Medicine, University of Toyama, 2630 Sugitani, Toyama, 930-0194 Japan

## Abstract

We investigated the role of the PI3K p85α subunit in the development of acute colitis with a focus on intestinal macrophages. Experimental acute colitis was induced using 3% dextran sulfate sodium (DSS) in drinking water for 7 days. The severity of DSS-induced acute colitis was significantly attenuated in p85α hetero-deficient (p85α+/−) mice compared with WT mice. The expression of proinflammatory mediators in intestinal macrophages isolated from the inflamed colonic mucosa was significantly suppressed in p85α+/− colitis mice compared with WT colitis mice. Interestingly, we found that bone marrow-derived macrophages (BMDMs) from p85α+/− mice produced a significantly higher amount of IL-10 than BMDMs from WT mice. The adoptive transfer of p85α+/− BMDMs, but not WT BMDMs, significantly improved the severity in WT colitis mice, and this effect was reversed by anti-IL-10 antibody. Furthermore, the expression of IL-10 in the intestinal macrophages of p85α+/− normal colonic mucosa was significantly higher than that in the intestinal macrophages of WT normal colonic mucosa. The present results demonstrate that p85α+/− mice exhibit a reduced susceptibility to DSS-induced acute colitis. Our study suggests that a deficiency of PI3K p85α enhances the production of IL-10 in intestinal macrophages, thereby suppressing the development of DSS-induced acute colitis.

## Introduction

Inflammatory bowel disease (IBD), Crohn’s disease and ulcerative colitis are chronic inflammatory disorders^1^. IBD has long been recognized to have a genetic basis, and likely involves a response of the immune system to some environmental agents. Abnormalities of intestinal innate immune functions and their relationship to the microbiota have been identified as key properties that characterize the immunogenetic profile of IBD and animal IBD models^1^. PI3Ks have important functions in the innate immune system^2^. Class IA PI3Ks are a family of heterodimeric enzymes consisting of a regulatory subunit (p85α, p55α, p50α, p85β or p55γ) and a catalytic subunit (p110α, p110β or p110δ)^3^. PI3K p85α is the most abundantly expressed among the regulatory subunits and is crucial for the development and functions of various innate immune cells such as dendritic cells^4^, macrophages^5^ and mast cells^6^. Thus, p85α-deficient mice showed impaired bacterial clearance in response to acute septic peritonitis^6^ and failed to develop a food allergy, which were attributed to a deficiency of mast cells in the intestine^7^. However, the role of the p85α subunit in IBD remains unclear, although several reports have revealed that PI3K plays important roles in the pathogenesis of colitis in humans or mice^8, 9^.

Recently, intestinal macrophages and dendritic cells have been identified as key regulators of immune homeostasis and inflammation in the intestine^10^. Resident intestinal macrophages can regulate themselves and other immune cells primarily through the spontaneous secretion of IL-10 that ultimately contributes to the prevention of pathological intestinal inflammation^11, 12^. In contrast, intestinal macrophages in the inflamed mucosa respond to microbial stimulation and produce large amounts of proinflammatory cytokines that further induce inflammation and damage in the intestine^13, 14^.

In the present study, we investigated the role of PI3K p85α in murine acute colitis, focusing on the cytokine production of macrophages. We demonstrated that p85α hetero-deficient (p85α+/−) mice exhibited a reduced susceptibility to dextran sulfate sodium (DSS)-induced acute colitis through an enhancement of IL-10 production in the intestinal macrophages, and high IL-10-producing macrophages protected the mice from the development of DSS-induced acute colitis.

## Results

### p85α+/− mice show a reduced susceptibility to DSS-induced acute colitis

**Figure 1:**
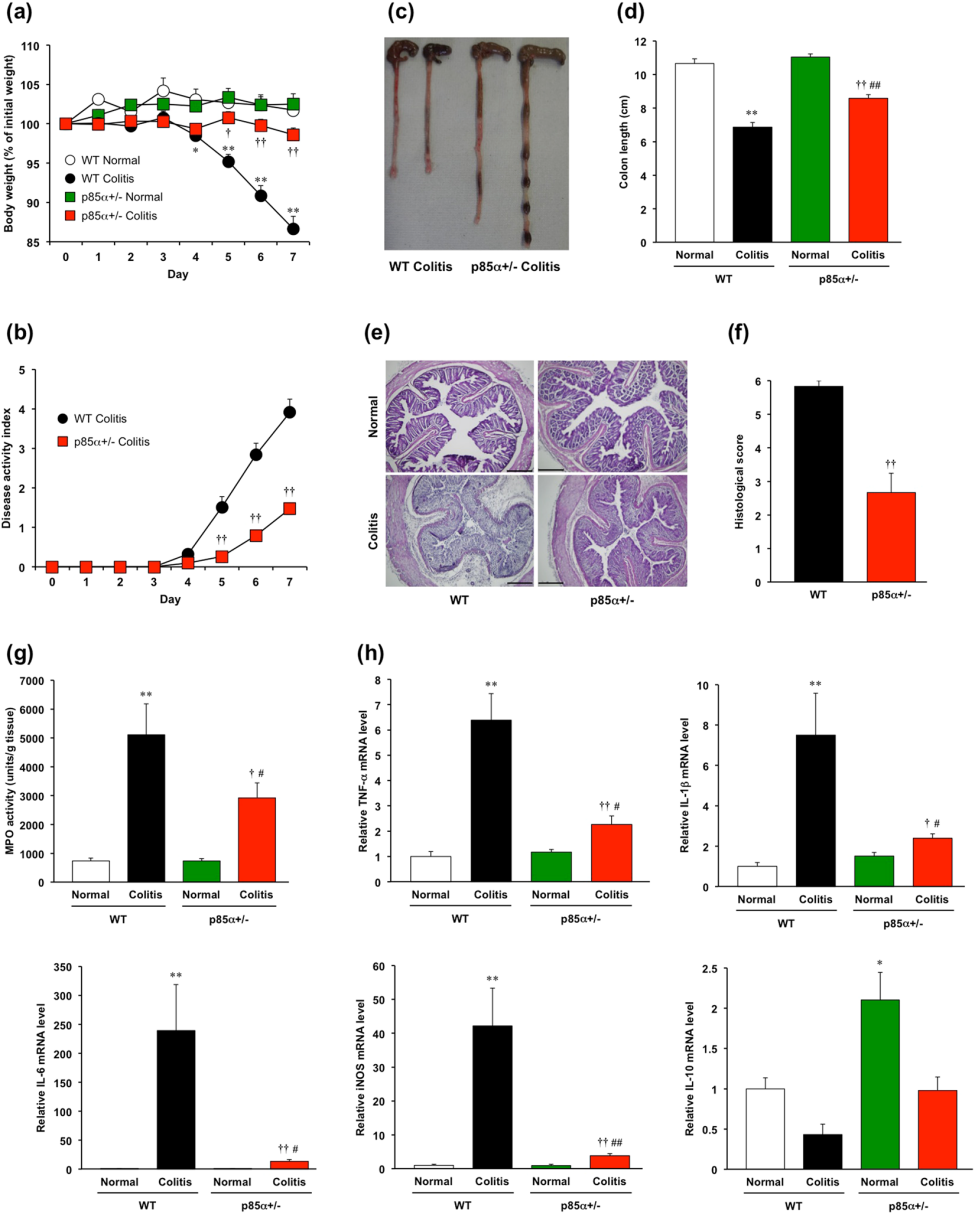
p85α+/− mice show reduced susceptibility to DSS-induced acute colitis. Colitis was induced in WT or p85α+/− mice through daily treatment with a 3% DSS solution in drinking water for 7 days. (**a**) Body weight, (**b**) disease activity index, (**c**) macroscopic observations of colons and (**d**) colon length are shown. (**e**) Representative images of H&E staining are shown. Scale bar is 300 μm. (**f**) Histological scoring of DSS-induced colitis. (**g**) Myeloperoxidase activity induced by DSS treatment in the mouse colons of WT or p85α+/− mice. (**h**) The changes in the mRNA expression of proinflammatory mediators and IL-10 induced by DSS treatment in the mouse colons of WT or p85α+/− mice. The data are presented as the mean ± SE of 4–6 mice and are representative of 1 out of 3 independent experiments. *p < 0.05; **p < 0.01, compared with WT normal mice. ^†^p < 0.05; ^††^p < 0.01, compared with WT colitis mice. ^#^p < 0.05; ^##^p < 0.05, compared with p85α+/− normal mice.

To clarify the role of the p85α subunit in colitis, we examined the development of DSS-induced acute colitis in WT and p85α+/− mice. Body weight loss was observed on day 4 after the start of DSS treatment in WT colitis mice and was significantly attenuated in p85α+/− colitis mice (Fig. 1a; 86.6 ± 1.6% in WT colitis, 98.6 ± 0.9% in p85α+/− colitis at day 7). The score of the disease activity index, which was a combination of diarrhea and rectal bleeding, was significantly alleviated in p85α+/− colitis mice compared with WT colitis mice (Fig. 1b; 3.9 ± 0.3 in WT, 1.5 ± 0.1 in p85α+/− at day 7). Macroscopic observations showed that the shortening of the colon caused by DSS treatment was significantly attenuated in p85α+/− colitis mice compared with WT colitis mice (Fig. 1c and d), although the colon length under normal conditions was similar between WT and p85α+/− mice (Fig. 1d). As shown in Fig. 1e and f, H&E staining of the colon from DSS-induced WT colitis mice revealed a loss of epithelial integrity and crypt architecture as well as submucosal edema, which were significantly improved in p85α+/− colitis mice. The MPO activity in the colons of WT colitis mice was markedly elevated on day 7 (Fig. 1g). The elevated MPO activity was significantly suppressed in the p85α+/− colitis mice. Furthermore, the expression of proinflammatory mediators such as TNF-α, IL-1β, IL-6 and iNOS mRNA in the colons of WT colitis mice was markedly upregulated on day 7 and significantly higher than those of p85α+/− colitis mice (Fig. 1h). We observed that the expression of IL-10 mRNA in the colons of p85α+/− mice was higher than those of WT mice in both normal and colitis states (Fig. 1h). These results clearly demonstrate that the development of DSS-induced colitis is suppressed in p85α+/− mice compared with WT mice.

To examine the involvement of PI3K activation in the colitis, we evaluated the effects of the PI3K inhibitors LY294002 and wortmannin on the development of DSS-induced colitis. LY294002 (1 mg/kg) and wortmannin (0.1 mg/kg) aggravated the body weight loss and disease activity index caused by DSS treatment in WT mice (Figure S1).

### Intestinal macrophages of p85α+/− mice express low levels of proinflammatory mediators

**Figure 2:**
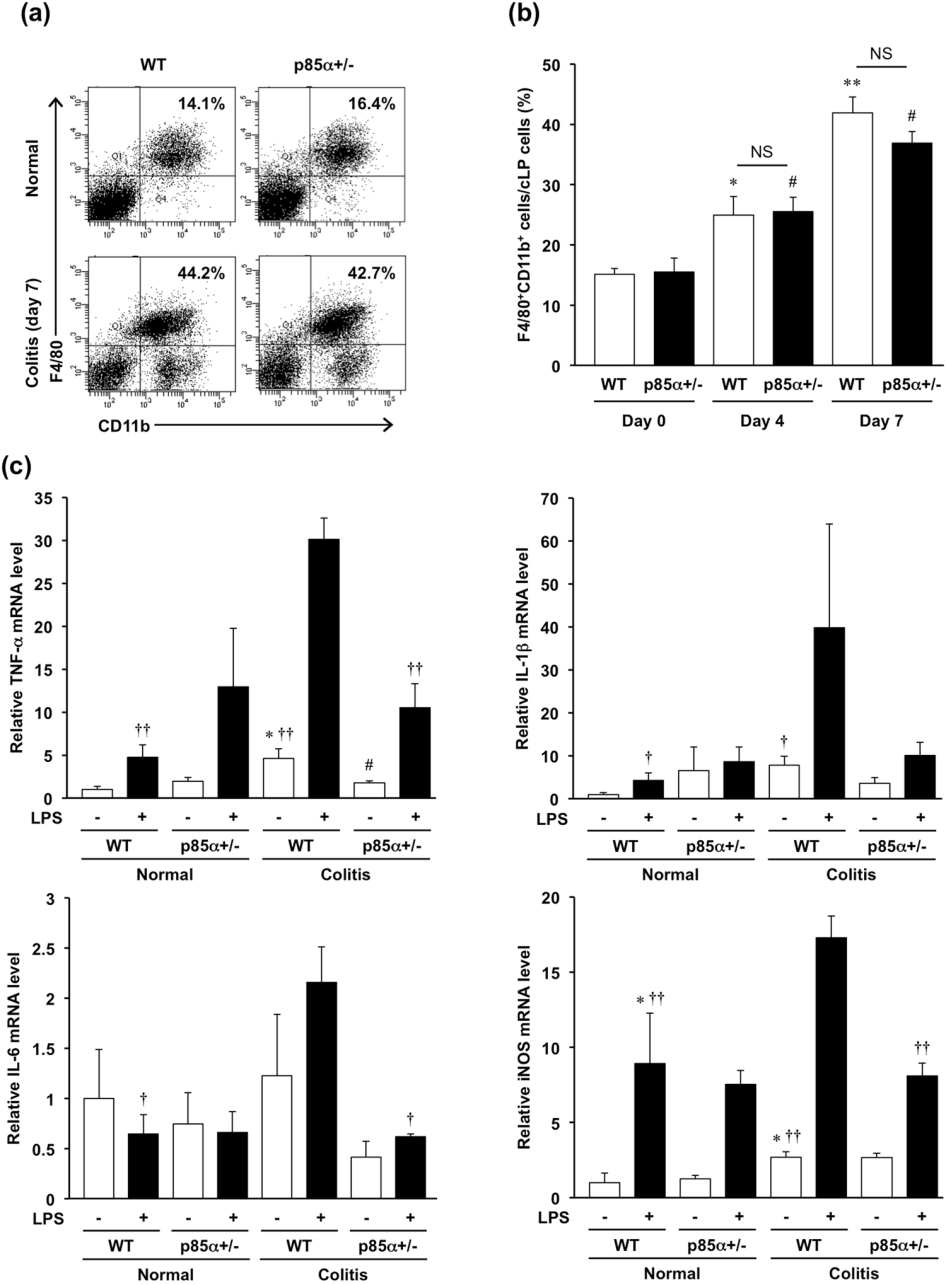
Intestinal macrophages in p85α+/− colitis mice express low levels of proinflammatory mediators. Colitis was induced in WT or p85α+/− mice through daily treatment with a 3% DSS solution in drinking water for 7 days. cLP cells were isolated from the colons in WT or p85α+/− mice at day 0 (normal), day 4 or day 7 after the start of DSS treatment. (**a**) Representative flow cytometry plots show the proportion of F4/80^+^CD11b^+^ intestinal macrophages in cLP cells. Plots are representative of the results from 4 independent experiments. (**b**) Quantification of the percentage of F4/80^+^CD11b^+^ macrophages in cLP cells was performed using flow cytometry analysis. *p < 0.05; **p < 0.01, compared with WT normal (Day 0) mice. ^#^p < 0.05, compared with p85α+/− normal (Day 0) mice. (**c**) Proinflammatory mediator mRNA expression in F4/80^+^ macrophages purified from the cLP cells of WT or p85α+/− mice at day 0 (normal) or day 7 using the IMag system. F4/80^+^ macrophages were stimulated with LPS (100 ng/ml) for 4 h. The data are presented as the mean ± SE of 4 independent experiments. *p < 0.05, compared with WT normal LPS (−). ^†^p < 0.05; ^††^p < 0.01, compared with WT colitis LPS (+). ^#^p < 0.05, compared with WT colitis LPS (−).

We examined the proportion of F4/80^+^CD11b^+^ macrophages in the colonic lamina propria. There was no difference in the proportion of the intestinal macrophages between WT and p85α+/− normal mice (Fig. 2a and b). The proportion of the intestinal macrophages was increased equivalently in both WT and p85α+/− mice in response to DSS treatment (Fig. 2a and b).

To investigate the expression levels of proinflammatory mediators in the intestinal macrophages, we isolated F4/80^+^ macrophages from the colonic lamina propria of mice on day 0 (normal) or 7 (colitis) after the start of DSS treatment. The intestinal macrophages of WT colitis mice expressed high levels of TNF-α, IL-1β, IL-6 and iNOS mRNA compared with the intestinal macrophages of WT normal mice (Fig. 2c). In contrast, the intestinal macrophages of p85α+/− colitis mice expressed similar levels of TNF-α, IL-1β, IL-6 and iNOS mRNA compared with intestinal macrophages of p85α+/− normal mice (Fig. 2c). Furthermore, the expression levels of these mediators in the intestinal macrophages of p85α+/− colitis mice were significantly lower than those in the intestinal macrophages of WT colitis mice (Fig. 2c). These results suggest that the p85α subunit is involved in the development of DSS-induced acute colitis by regulating the production of proinflammatory mediators in the intestinal macrophages of the inflamed colonic mucosa.

We also examined the proportion of Ly6C^+^CD11b^+^ inflammatory monocytes and Gr1^+^CD11b^+^ neutrophils in the colonic lamina propria. There was no difference in the proportion of intestinal inflammatory monocytes and neutrophils between WT and p85α+/− colitis mice (Figure S2A and B).

### Bone marrow-derived macrophages from p85α+/− mice produce a large amount of IL-10

**Figure 3:**
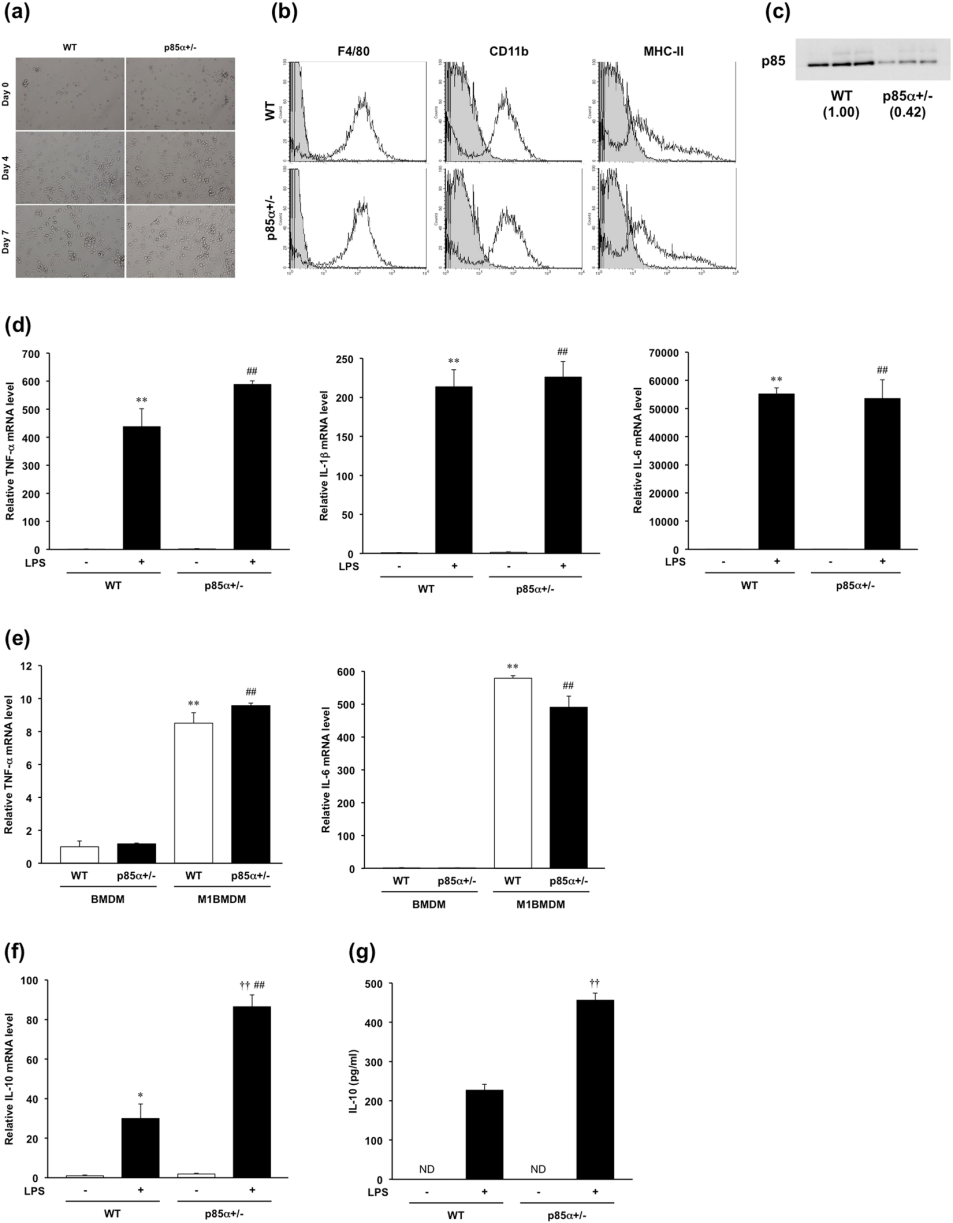
p85α-deficient macrophages produce a large amount of IL-10. BMDMs were prepared from the femurs and tibiae of WT or p85α+/− mice and cultured with M-CSF (100 ng/ml) for 7 days. (**a**) Representative images of BMDM morphology. (**b**) Representative flow cytometry histogram. (**c**) Representative images of PI3K p85α protein expression in WT or p85α+/− BMDMs as detected by western blotting. (**d**) Proinflammatory mediator mRNA expression in BMDMs from WT or p85α+/− mice. BMDMs were stimulated with LPS (100 ng/ml) for 4 h. (**e**) Proinflammatory cytokine mRNA expression in M1BMDMs from WT or p85α+/− mice. (**f**) IL-10 mRNA expression in BMDMs from WT or p85α+/− mice. BMDMs were stimulated with LPS (100 ng/ml) for 4 h. (**g**) The concentration of IL-10 in the culture supernatants of BMDMs. BMDMs were stimulated with LPS (100 ng/ml) for 24 h, and the culture supernatant was analyzed for IL-10 protein using a CBA kit. The data are presented as the mean ± SE of 4 independent experiments. *p < 0.05; **p < 0.01, compared with WT BMDM LPS (−). ^††^p < 0.01, compared with WT BMDM LPS (+). ^##^p < 0.01, compared with p85α+/− BMDM LPS (−).

To investigate the contribution of the p85α subunit to the cytokine production of macrophages, we screened the expression levels of cytokine mRNA in BMDMs from WT or p85α+/− mice. We observed no morphological changes between WT and p85α+/− BMDMs (Fig. 3a). Furthermore, there was no difference in the proportion of F4/80^+^, CD11b^+^ or MHC-II^+^ cells between WT and p85α+/− BMDMs (Fig. 3b). The protein expression of PI3K p85α in p85α+/− BMDMs was less than 50% compared to WT BMDMs (Fig. 3c). The mRNA expression of proinflammatory cytokines in p85α+/− BMDMs was similar to WT BMDMs (Fig. 3d). To examine the mRNA expression of proinflammatory cytokines in inflammatory (M1) macrophages, BMDMs were further stimulated with IFN-γ and LPS for 24 h and were polarized to M1BMDMs. However, there was no difference in the expression of proinflammatory cytokine mRNAs between WT and p85α+/− M1BMDMs, although both M1BMDMs showed marked upregulation of proinflammatory cytokine expression compared with BMDMs (Fig. 3e). Interestingly, a significant threefold increase in IL-10 mRNA expression was observed in p85α+/− BMDMs compared with WT BMDMs (Fig. 3f). Furthermore, p85α+/− BMDMs secreted a significantly higher amount of IL-10 in response to LPS stimulation than WT BMDMs (Fig. 3g). The BMDMs from p85α KO mice also showed a significant threefold increase in IL-10 mRNA expression compared with those from WT mice (Figure S3).

**Figure 4:**
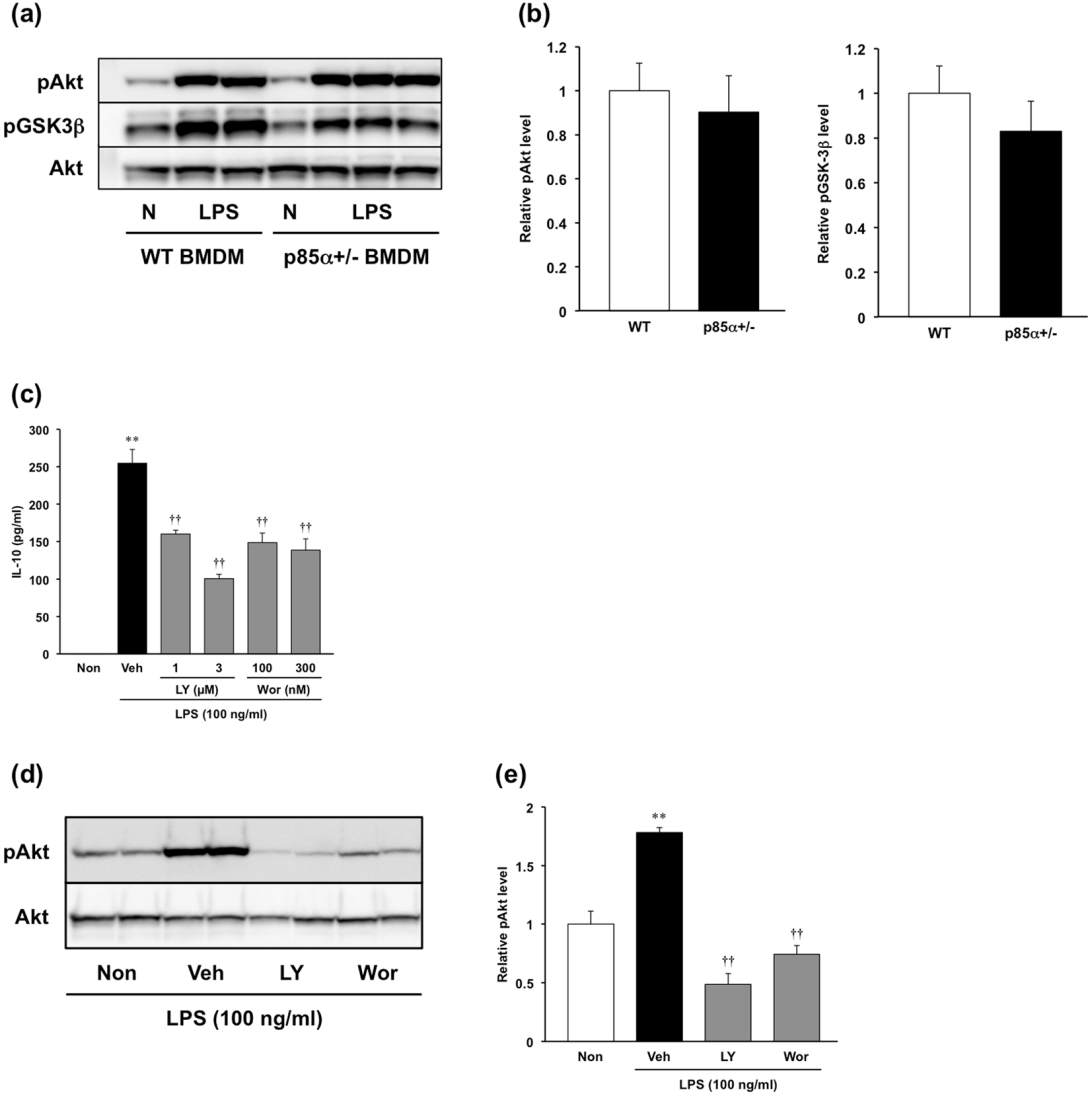
The production of a large amount of IL-10 in p85α+/− BMDMs is independent of Akt activation. BMDMs were prepared from the femurs and tibiae of WT or p85α+/− mice and cultured with M-CSF (100 ng/ml) for 7 days. (**a**) Representative images of pAkt and pGSK-3β protein level in WT or p85α+/− BMDMs as detected by western blotting. BMDMs were stimulated with LPS (100 ng/ml) for 1 h. (**b**) Relative pAkt and pGSK-3β levels were normalized by Akt signals. (**c**) Effect of LY294002 and wortmannin on the concentration of IL-10 in the culture supernatant of WT BMDMs. LY294002 (1–3 μM) and wortmannin (100–300 nM) was applied 30 min before LPS stimulation. (**d**) Representative images of pAkt protein level in LY294002 (LY) or wortmannin (Wor)-treated BMDMs as detected by western blotting. (**e**) Relative pAkt levels were normalized by Akt signals. The data are presented as the mean ± SE of 4 independent experiments. **p < 0.01, compared with non-stimulation (Non). ^††^p < 0.01, compared with vehicle (Veh).

Next, we examined the expression of phosphorylated Akt in WT and p85α+/− BMDMs. Immunoblot analysis revealed that phosphorylated Akt was equivalently upregulated by LPS stimulation in both WT and p85α+/− BMDMs (Fig. 4a and b). Furthermore, there was no difference in the level of phosphorylated GSK-3β between WT and p85α+/− BMDMs (Fig. 4a and b). To clarify the involvement of Akt activation in the regulation of IL-10 production, we examined the effects of LY294002 and wortmannin on IL-10 secretion in WT BMDMs. LY294002 (1–3 μM) and wortmannin (100–300 nM) significantly inhibited the LPS-stimulated IL-10 secretion of WT BMDMs in a concentration-dependent manner (Fig. 4c). The expression of phosphorylated Akt induced by LPS stimulation was significantly suppressed by treatment with LY294002 and wortmannin (Fig. 4d and e). These results suggest that p85α+/− BMDMs produce a large amount of IL-10 through an Akt-independent pathway.

**Figure 5:**
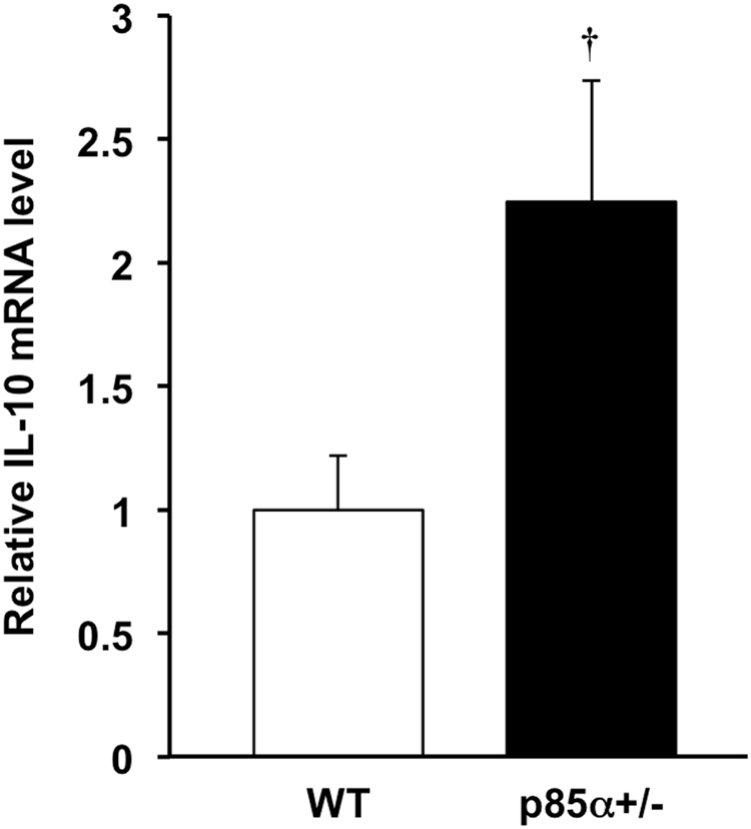
Intestinal macrophages from p85α+/− normal mice express a high level of IL-10 mRNA. F4/80^+^ macrophages were isolated from the colons of normal WT or p85α+/− mice using the IMag system. F4/80^+^ macrophages were stimulated with LPS (100 ng/ml) for 4 h. The data are presented as the mean ± SE of 4 independent experiments. ^†^p < 0.05, compared with WT mice.

Furthermore, we investigated the IL-10 mRNA expression of intestinal macrophages in the normal colonic mucosa. In the steady state, the intestinal macrophages of p85α+/− mice showed a significant increase in IL-10 mRNA expression compared with those of WT mice (Fig. 5). These findings suggest that the intestinal macrophages in p85α+/− mice suppress the development of acute colitis through a high production of IL-10 in colonic mucosa.

### High IL-10-producing bone marrow-derived macrophages suppress the development of DSS-induced acute colitis

**Figure 6:**
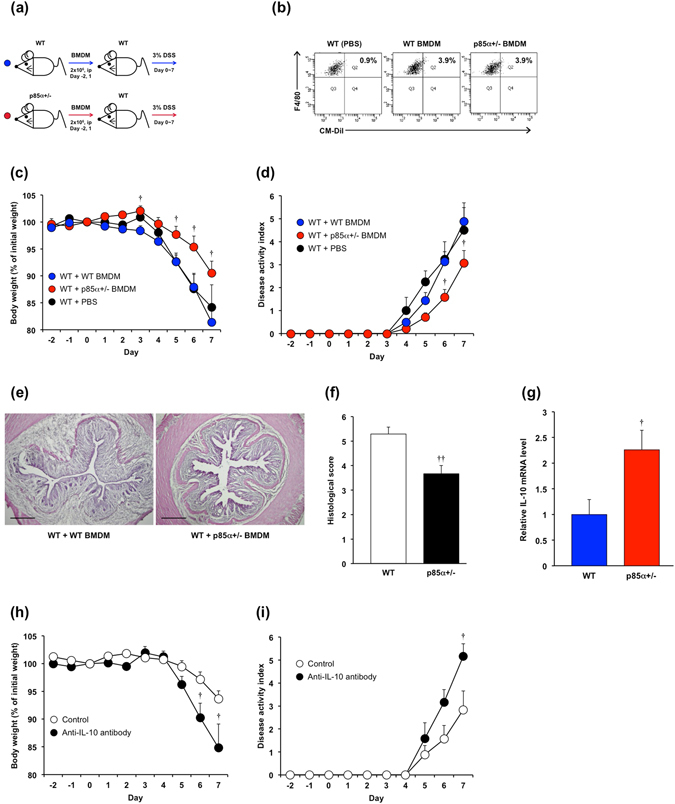
High IL-10-producing macrophages suppress the development of DSS-induced acute colitis in WT mice. (**a**) Schematic drawing of the experimental design for the evaluation of BMDM adoptive transfer in the DSS-induced acute colitis model. BMDMs from WT or p85α+/− mice were intraperitoneally injected into WT mice at 2 days before and 1 day after the start of DSS treatment. (**b**) Representative flow cytometry plots show the proportion of CD11b^+^F4/80^+^CM-DiI^+^ intestinal macrophages in cLP cells. Plots were representative of the results from 3 independent experiments. Body weight (**c**), disease activity index (**d**) and representative images of H&E staining (**e**) are shown. Scale bar is 300 μm. (**f**) Histological scoring of DSS-induced colitis. (**g**) IL-10 mRNA expression in the mouse colons of WT BMDM or p85α+/− BMDM-transferred colitis mice. The data are presented as the mean ± SE of 4 mice and are representative of 1 out of 3 independent experiments. ^†^p < 0.05; ^††^p < 0.01, compared with WT BMDM. (**h**,**i**) Effect of anti-IL-10 antibody on the ameliorative action of p85α+/− BMDM against the development of DSS-induced colitis in WT mice. Anti-IL-10 antibody or control IgG was injected intraperitoneally into p85α+/− BMDM-transferred WT mice 2 h before the start of DSS treatment and then every other day. Body weight (**h**) and disease activity index (**i**) are shown. The data are presented as the mean ± SE of 5 mice and are representative of 1 out of 2 independent experiments. ^†^p < 0.05, compared with Control.

To investigate whether high IL-10-producing macrophages can suppress the development of acute colitis, BMDMs from WT or p85α+/− mice were injected into WT mice in a DSS-induced colitis model (Fig. 6a). Adoptively transferred BMDMs from each genotype were equivalently located in the colonic lamina propria at 2 days after the injection (Fig. 6b; 4.2 ± 0.2% in WT BMDM, 4.6 ± 0.3% in p85α+/− BMDM). Adoptive transfer of p85α+/− BMDMs, but not WT BMDMs, significantly suppressed the body weight loss and disease activity index caused by DSS treatment in WT mice (Fig. 6c and d). Histological analysis also showed that pathological abnormalities were improved in p85α+/− BMDM-transferred colitis mice compared with WT BMDM-transferred colitis mice (Fig. 6e and f). The expression of IL-10 mRNA was significantly higher in the colons of p85α+/− BMDM-transferred colitis mice than those of WT BMDM-transferred colitis mice (Fig. 6g). Furthermore, the ameliorative effect of p85α+/− BMDM transfer was significantly reversed by the administration of anti-IL-10 antibody (Fig. 6h and i). These results demonstrate that high IL-10-producing p85α+/− BMDMs suppress the development of DSS-induced acute colitis.

## Discussion

Several studies have reported a role for PI3K in colitis in humans and animal models. The activation of PI3K/Akt signaling has been observed in the colonic mucosal biopsy specimens of ulcerative colitis patients and DSS-induced colitis model^8^. A deficiency in the PI3K p110δ subunit in mice develops spontaneous colitis because the p110δ subunit controls homeostatic antigen presenting cell-T cell interactions by altering the balance between IL-10 and IL-12/23^9, 15^. Furthermore, p110δ-deficient mice show a reduction in the number of arginase I-expressing M2 macrophages in the colonic mucosa, resulting in the development of more severe DSS-induced colitis than WT mice^16^. Additionally, an inhibitor of PI3Kγ has been reported to suppress the development of murine colitis^17, 18^. These studies indicate that PI3K subunits have roles in the pathogenesis of colitis. Here, we report that mice deficient in the p85α subunit exhibit a reduced susceptibility to DSS-induced acute colitis due to the augmentation of IL-10 production by intestinal macrophages in the colonic mucosa.

It is generally accepted that innate immunity largely contributes to the pathogenesis of DSS-induced acute colitis^19^. In particular, enhanced infiltration and activation of macrophages in the colonic mucosa play a crucial role in the inflammatory response^13^. Although we also found that the proportion of intestinal macrophages was increased in the inflamed colonic mucosa after DSS treatment as previously reported^13^, there was no difference between WT and p85α+/− colitis mice. Furthermore, we did not observe a difference in the proportion of Ly6C^+^CD11b^+^ inflammatory monocytes and Gr1^+^CD11b^+^ neutrophils (Supplemental Figure S2A and B), which infiltrate the colonic mucosa after DSS treatment and are involved in the pathogenesis of DSS-induced colitis^20, 21^. Therefore, our findings suggest that a deficiency of p85α has no effect on the infiltration of proinflammatory innate immune cells in DSS-induced colitis.

Inflammatory macrophages in the inflamed colonic mucosa produce a large amount of proinflammatory mediators such as TNF-α, IL-1β, IL-6 or iNOS^22^. In IBD patients, inflammatory macrophages in the inflamed colonic mucosa cause the expansion of further inflammation through a large amount of proinflammatory cytokine production^23^. We demonstrated that the intestinal macrophages that were isolated from inflamed colonic mucosa of p85α+/− colitis mice expressed low levels of proinflammatory mediators compared with those of WT colitis mice. We assumed that the p85α subunit also contributed to the production of proinflammatory cytokines in macrophages because the p85α subunit in BMDMs is crucial for proliferation and migration^5^.

Monocytes recruited by CCR2 differentiate locally into intestinal macrophages through stimulation of M-CSF^24^. Thus, M-CSF-induced BMDMs are widely used as an *in vitro* model for intestinal macrophages^25^. Furthermore, the stimulation of BMDMs *in vitro* with INF-γ and LPS generates M1BMDMs, which exhibit the features of proinflammatory macrophages^26^. Our results indicated that p85α deficiency did not influence the expression of proinflammatory mediators in both BMDMs and M1BMDMs. Interestingly, p85α-deficient BMDMs produced a higher amount of IL-10 than WT BMDMs. Resident intestinal macrophages express low levels of Toll-like receptors and do not produce proinflammatory cytokines after exposure to bacterial signals^11, 25, 27^. In mice, this state of inflammatory anergy is largely attributable to IL-10 that is constitutively expressed by intestinal macrophages^10^. A lack of intestinal macrophages resulted in the enhanced severity of DSS-induced acute colitis^28, 29^, which was reversed by the adoptive transfer of BMDMs^28^. Intestinal macrophages have also been reported to suppress the development of DSS-induced colitis due to the enhanced release of IL-10 from intestinal macrophages by treatment with *Clostridium butyricum* ^30^. These reports suggest that p85α deficiency augments IL-10 production in intestinal macrophages, thereby suppressing the development of DSS-induced acute colitis in p85α+/− mice. Indeed, intestinal macrophages of p85α+/− mice expressed a high level of IL-10 in the steady state. A noteworthy finding in the current study was that the adoptive transfer of high IL-10-producing macrophages (p85α+/− BMDMs) significantly suppressed the development of DSS-induced acute colitis, and this effect was impaired by anti-IL-10 antibody. The injection of anti-IL-10 antibody did not affect the development of DSS-induced colitis in WT BMDM-transferred WT mice (Figure S4A and B), suggesting that anti-IL-10 antibody has no profound pro-inflammatory effect. We also verified that the intraperitoneally injected BMDMs were distributed in the colonic lamina propria in a proportion equivalent to that in a previous report^31^. These results strongly indicate that p85α deficiency in mice enhances the IL-10 production of intestinal macrophages, resulting in the suppression of acute colitis development.

Akt phosphorylation has widely been used as an indicator of PI3K activity because Akt is thought to be the direct downstream target of PI3K^32^. A previous report has shown that the phosphorylation of Akt that is induced by M-CSF stimulation is decreased in BMDMs derived from p85α KO mice^5^. However, the present study demonstrated that no difference in Akt and GSK-3β phosphorylation was induced by LPS stimulation between WT and p85α+/− BMDMs. Furthermore, we observed that PI3K inhibitor treatment at the effective concentration for the inhibition of Akt activation reduced IL-10 production in BMDMs, which is consistent with previous finding^33^. An inhibition of GSK-3β has also been reported to accelerate the LPS-induced IL-10 production^34^. A recent study has revealed that the specific interaction between p85α and intracellular osteopontin is essential for the sustained expression of Bcl-6 and the functional differentiation of follicular T cells^35^, implicating the role of a p110-Akt-independent pathway in p85α+/− mice. In the current study, p85α KO BMDMs also produced similar IL-10 levels as p85α+/− BMDMs, suggesting that the heterodeficiency of p85α is sufficient for the enhancement of IL-10 in macrophages. Thus, the augmentation of IL-10 production in p85α+/− macrophages is independent of PI3K/Akt/GSK-3β signaling, although further studies are needed to clarify the detailed mechanisms.

IL-10 plays a pivotal role in the regulation of intestinal homeostasis during host defense^36^. The association between IL-10 and IBD has been demonstrated by several findings in both humans and animal models. Mutations in genes encoding the IL-10 receptor subunit were found in patients with severe early-onset IBD^26, 37^. Furthermore, genome-wide association studies have identified single nucleotide polymorphisms in IL-10 that are associated with a susceptibility to Crohn’s disease and ulcerative colitis^38^. Consistent with human IBD, the development of spontaneous colitis has been observed in mice with the IL-10 receptor^39^ or STAT3^40^ deletion in macrophages as well as IL-10-deficient mice^41^. Subcutaneous recombinant IL-10 treatment was examined as a potential therapeutic in IBD patients in a clinical trial, but it was not effective^42, 43^. The systemic administration of IL-10 does not work efficiently for a sufficient period at local sites of intestinal inflammation because recombinant IL-10 may be cleared before reaching its target due to the short half-life of IL-10^44^. Actually, localized delivery of IL-10-secreting *Lactococcus lactis* ameliorated DSS-induced chronic colitis and the onset of colitis in IL-10-deficient mice^45^. Thus, our results suggest that the acceleration of IL-10 production by intestinal macrophages in the colonic mucosa may be beneficial for suppressing intestinal inflammation in IBD patients.

Given the findings of the present study, we conclude that p85α+/− intestinal macrophages protect mice from the development of DSS-induced acute colitis via the enhancement of IL-10 production in the cLP. In addition, the augmentation of IL-10 production in intestinal macrophages has the potential to be a novel therapeutic target for IBD.

## Materials and Methods

All experiments were performed in accordance with the Guide for the Care and Use of Laboratory Animals of the National Institutes of Health and the University of Toyama. The Animal Experiment Committee at the University of Toyama approved all of the animal care procedures and study protocols (authorization no. A2012INM-2 and A2015INM-2).

### Mice

p85α-deficient mice on a BALB/c background^46^ were gifted from Drs. Shigeo Koyasu (RIKEN Center for Integrative Medical Sciences, Yokohama, Japan) and Takashi Kadowaki (The University of Tokyo, Tokyo, Japan). Male BALB/c mice were purchased from Japan SLC (Shizuoka, Japan). All mice were housed in the experimental animal facility at the University of Toyama and were provided free access to food and water.

### DSS-induced acute colitis model

Mice were treated with 3% DSS (36–50 kDa; MP Biomedicals, Santa Ana, CA) in their drinking water for 7 days^47^. To assess the severity of colitis, body weight, stool consistency, and blood in the stool were monitored daily. The disease activity index was the sum of 2 parameters: diarrhea (0, normal; 1, loose stools; 2, watery diarrhea) and blood in the stool (0, normal; 1, slight bleeding; 2, gross bleeding). The researcher measuring the disease activity index was blinded to the mouse group.

### Histological study

The distal part of the colon was removed, washed with ice-cold phosphate-buffered saline, and immersed in 4% paraformaldehyde for 24 h at 4 °C. After treatment with a 30% sucrose solution, the tissue sample was embedded in Tissue Freezing Medium. Frozen sections (10 μm) were cut at −20 °C using a cryostat microtome (Leica Microsystems, Nussloch, Germany). The sections were then routinely stained with H&E. H&E-stained sections were scored for inflammation and crypt damage as described previously^47^. To exclude bias, histological scores were determined in a masked manner.

### Determination of myeloperoxidase activity

Myeloperoxidase (MPO) activity was measured in the mouse colon as described previously^47^. Briefly, the animals were sacrificed 7 days after DSS treatment, and the colons were excised. After the tissue was rinsed with ice-cold saline, the whole colon was weighed and homogenized on ice in 50 mM phosphate buffer containing 0.5% hexadecyltrimethylammonium bromide (pH 6.0). The homogenized samples were subjected to 3 cycles of freeze-thawing and then centrifuged at 2,000 g for 10 min at 4 °C. The MPO activity in the supernatant was determined by adding the supernatant to 0.5 M *o*-dianisidine hydrochloride in 10 mM phosphate buffer (pH 6.0) containing 0.00005% (wt/vol) hydrogen peroxide. The changes in the absorbance of each sample were recorded at 460 nm using a spectrophotometer (UV160A; Shimadzu, Kyoto, Japan). MPO activity was expressed as units per wet weight of colonic tissue in grams.

### Determination of mRNA expression

Cytokine mRNA expression was measured in the mouse colon as described previously^47^. Briefly, total RNA was extracted from the colon using Sepasol RNA I Super (Nacalai Tesque, Kyoto, Japan) according to the manufacturer’s instructions. Reverse transcription was performed using the PrimeScript RT reagent Kit (Takara Bio, Ohtsu, Japan) and random primers followed by real-time PCR. Real-time PCR amplification of TNF-α, IL-1β, IL-6, IL-10, iNOS, and GAPDH was performed using SYBR Premix EX Taq (Takara Bio). The following primer pairs were used: TNF-α, (forward) 5′-AAGCCTGTAGCCCACGTCGTA-3′ and (reverse) 5′-GGCACCACT- AGTTGGTTGTCTTTG-3′; IL-1β, (forward) 5′-CTGTGTCTTTCCCGTGGACC-3′ and (reverse) 5′-CAGCTCATATGGGTCCGACA-3′; IL-6, (forward) 5′-CCACTT-CACAAGTCGGAGGCTTA-3′ and (reverse) 5′-GCAAGTGCATCATCGTTGTTC-ATAC-3′; IL-10, (forward) 5′-GGCCCTTTGCTATGGTGTCC-3′ and (reverse) 5′-AAGCGGCTGGGGGATGAC-3′; iNOS, (forward) 5′-TCCTGGAGGAAGTGG-GCCGAAG-3′ and (reverse) 5′-CCTCCACGGGCCCGGTACTC-3′; GAPDH, (forward) 5′-TGACCACAGTCCATGCCATC-3′ and (reverse) 5′-GACGGACAC-ATTGGGGGTAG-3′. Real-time PCR was performed using the Takara TP800 (Takara Bio). The PCR reaction conditions consisted of 10 s at 95 °C followed by 40 cycles of 5 s at 95 °C and 20 s at 60–63 °C. Target mRNA levels were normalized to those of GAPDH as an internal control in each sample. The results are expressed as ratios relative to the average of the control group.

### Isolation of lamina propria macrophages

Colonic lamina propria (cLP) cells were isolated from the mouse colons as described previously^47^. Briefly, the colons were removed, opened longitudinally, and washed of fecal contents with ice-cold RPMI-1640 (Wako, Osaka, Japan). The colons were then cut into small pieces that were stirred at 37 °C for 20 min in RPMI-1640 containing 2% FBS (GIBCO, Carlsbad, CA) and 0.5 mM EDTA and washed twice with RPMI-1640. This process was repeated without EDTA. The pieces were incubated at 37 °C for 20 min in RPMI-1640 containing 200 U/ml collagenase (Wako), and the digested tissues were collected and washed with RPMI-1640. This process was repeated 3 times, and cells were pooled. The pooled cell suspension was passed through a strainer (70 μm), and washed with RPMI-1640. Isolated cells were suspended in 40% Percoll (Sigma-Aldrich, St. Louis, MO), layered onto 75% Percoll, and centrifuged at 770 g for 20 min. cLP cells were recovered from the Percoll interphase and washed twice with RPMI-1640. cLP cells were stained with APC-conjugated anti-F4/80 antibody (eBioscience, San Diego, CA), and F4/80 positive cLP macrophages were purified using the BD IMag APC Magnetic Particles (BD Biosciences, San Diego, CA). For determination of cytokine mRNA expression, isolated cLP macrophages (2 × 10^5^) were stimulated with 100 ng/ml LPS (Sigma-Aldrich) for 4 h, and total RNA was extracted from the intestinal macrophages using an RNeasy Mini kit (Qiagen, Valencia, CA). RT-PCR was performed as described above.

### Flow cytometry analysis

The cells were incubated with FcR Blocking Reagent (Miltenyi Biotec, Auburn, CA) and Via-Probe (BD Biosciences) for 5 min followed by staining with specific antibodies for 30 min at 4 °C. Flow cytometry analyses were conducted on a FACSCanto II (BD Biosciences), and the data were analyzed with CellQuest Pro (BD Biosciences). The antibodies used were APC, FITC or PE-conjugated mAbs against CD11b (3A33) from Beckman Coulter (Brea, CA), F4/80 (BM8) and MHCII (M5/114.15.2) from eBioscience.

### Bone marrow-derived macrophage culture

Bone marrow-derived macrophages (BMDMs) were prepared from the femurs and tibiae of p85α+/− or WT mice and cultured with M-CSF (R&D systems, Minneapolis, MN). Bone marrow cells were cultured in RPMI-1640 medium supplemented with 100 ng/ml M-CSF, 10% heat-inactivated FBS, 55 μM 2-mercaptoethanol (GIBCO), 50 U/ml penicillin, and 50 μg/ml streptomycin (Sigma-Aldrich) at 37 °C in a humidified 5% CO_2_ atmosphere. After 7 days, macrophage purity was examined using flow cytometry (FACSCanto II), and more than 90% of the adherent cells were CD11b and F4/80 positive. For determination of cytokine mRNA expression, BMDMs (5 × 10^5^) were stimulated with LPS (100 ng/ml) for 4 h, and total RNA was extracted from BMDMs using an RNeasy Mini kit. RT-PCR was performed as described above.

### Measurement of IL-10 protein

BMDMs (5 × 10^5^) were seeded on 24-well culture plates (BD Biosciences) in RPMI-1640 supplemented with 10% FBS, 50 U/ml penicillin, and 50 μg/ml streptomycin (Sigma-Aldrich), and stimulated with LPS (100 ng/ml) for 24 h at 37 °C in a humidified incubator with 5% CO_2_. In some experiments, LY-294002 (1–3 μM; Sigma-Aldrich) or wortmannin (100–300 nM; Sigma-Aldrich) was applied 30 min before the LPS stimulation. Culture supernatants were collected and stored at −80 °C until IL-10 measurement was performed. IL-10 concentrations in culture supernatants were detected using a Cytometric Beads Array Kit (BD Biosciences) according to the manufacturer’s instructions. Samples were analyzed with a FACSCanto II flow cytometer.

### Determination of protein level using western blotting

BMDMs were stimulated with 100 ng/ml LPS for 1 h at 37 °C. In some cases, LY-294002 (3 μM) or wortmannin (300 nM) was applied 30 min before the LPS stimulation. BMDMs were homogenized in lysis buffer (pH 7.4) as described previously^47^. The samples (20 μl/lane) were then subjected to electrophoresis on 10% SDS-polyacrylamide gels and transferred electrophoretically to PVDF membranes (Millipore, Billerica, MA). The membranes were incubated with rabbit anti-pAkt (Ser473) antibody, rabbit anti-Akt antibody, rabbit anti-p-GSK-3β (Ser9) antibody or rabbit anti-PI3 Kinase p85 antibody (Cell Signaling Technology, Denvers, MA) and treated with horseradish peroxidase-conjugated goat anti-rabbit IgG (Cell Signaling Technology). Immune complexes were visualized using an enhanced chemiluminescence detection system (GE Healthcare Japan, Tokyo, Japan) and photographed (ImageQuant LAS4000; GE Healthcare Japan). Akt and pAkt protein expression levels were determined densitometrically with ImageJ (NIH, Bethesda, MD).

### Adoptive transfer

BMDMs (2 × 10^6^) from WT or p85α+/− mice were intraperitoneally injected into WT mice 2 days before and 1 day after the start of DSS treatment. To neutralize IL-10, LEAF Purified anti-mouse IL-10 or LEAF Purified Rat IgG1κ (250 μg/mouse; Biolegend, San Diego, CA) was injected intraperitoneally into p85α+/− BMDM-transferred WT mice 2 h before the start of DSS treatment and then every other day. To trace the transferred BMDMs *in vivo*, BMDMs were labeled with the fluorescent membrane marker CM-DiI (Molecular Probes, Eugene, OR) according to the manufacturer’s instructions and injected into mice. After 2 days, cLP cells were isolated from the mouse colons and the proportion of the CD11b^+^F4/80^+^CM-DiI^+^ intestinal macrophages was analyzed using a FACSCanto II flow cytometer.

### Statistical analyses

The data are presented as the means ± SE. Statistical analyses were performed using repeated measures two-way ANOVA followed by Bonferroni’s multiple- comparison test, a one-way ANOVA followed by a Dunnett’s multiple-comparison test, nonparametric Mann-Whitney test or an unpaired (two-tailed) t-test. Values of p < 0.05 were considered to be significant.

## Electronic supplementary material

Supplementary Information
