# Supplementary material for: PI3K p85α Subunit-deficient Macrophages Protect Mice from Acute Colitis due to the Enhancement of IL-10 Production
Source: Sci Rep. 2017 Jul 21;7:6187. doi: 10.1038/s41598-017-06464-w (PMC5522489; doi:10.1038/s41598-017-06464-w)
Supplement: Supplementary file 1 — Supplementary Information [file 41598_2017_6464_MOESM1_ESM.doc]

**PI3K p85α Subunit-deficient Macrophages Protect Mice from Acute Colitis due to the Enhancement of IL-10 Production**

Shusaku Hayashi^*,†^, Takayuki Hamada^†^, Donald G. A. Zinsou, Momoe Oshiro, Kana Itoi, Takeshi Yamamoto, and Makoto Kadowaki

Division of Gastrointestinal Pathophysiology, Institute of Natural Medicine, University of Toyama, 2630 Sugitani, Toyama 930-0194, Japan

*hayashi@inm.u-toyama.ac.jp

^†^these authors contributed equally to this work

**Figure S1**

**A B**

Figure S1. PI3K inhibitors aggravate the development of DSS-induced acute colitis in mice. Colitis was induced in WT mice through daily treatment with a 3% DSS solution in drinking water for 7 days. LY294002 (1 mg/kg) or wortmannin (0.1 mg/kg) was administered ip one time daily for 7 days. The data are presented as the mean ± SE of 4-8 mice and are representative of 1 out of 2 independent experiments. Body weight (A) and disease activity index (B) are shown. †p<0.05; ††p<0.01, compared with Vehicle.

**Figure S2**

**A B**

Figure S2. The proportion of Ly6C^+^CD11b^+^ inflammatory monocytes and Gr1^+^CD11b^+^ neutrophils are equivalent. Quantification of the percentage of Ly6C^+^CD11b^+^ inflammatory monocytes (A) and Gr1^+^CD11b^+^ neutrophils (B) in cLP cells was performed using flow cytometry analysis. *p<0.05, compared with WT normal (Day 0) mice. #p<0.05, compared with p85α+/- normal (Day 0) mice. The data are presented as the mean ± SE of 4 mice and are representative of 1 out of 2 independent experiments.

**Figure S3**

Figure S3. p85α-deficient macrophages produce a large amount of IL-10. BMDMs were prepared from the femurs and tibiae of WT or p85α KO mice and cultured with M-CSF (100 ng/ml) for 7 days. IL-10 mRNA expression in BMDMs from WT or p85α KO mice. BMDMs were stimulated with LPS (100 ng/ml) for 4 h. The data are presented as the mean ± SE of 3 independent experiments. ††p<0.01, compared with WT BMDM LPS (+).

**Figure S4**

**A** **B**

Figure S4. Anti-IL-10 antibody does not affect the development of DSS-induced colitis in WT BMDM-transferred WT mice. Anti-IL-10 antibody or control IgG was injected intraperitoneally into WT BMDM-transferred WT mice 2 h before the start of DSS treatment and then every other day. Body weight (A) and disease activity index (B) are shown. The data are presented as the mean ± SE of 4 mice.
